# STK3 is a therapeutic target for a subset of acute myeloid leukemias

**DOI:** 10.18632/oncotarget.25238

**Published:** 2018-05-22

**Authors:** Aylin Camgoz, Maciej Paszkowski-Rogacz, Shankha Satpathy, Martin Wermke, Martin V. Hamann, Malte von Bonin, Chunaram Choudhary, Stefan Knapp, Frank Buchholz

**Affiliations:** ^1^ Universitäts KrebsCentrums Dresden, Medical Systems Biology, Medical Faculty, Technische Universität Dresden, Dresden, Germany; ^2^ The Novo Nordisk Foundation Center for Protein Research, University of Copenhagen, Copenhagen, Denmark; ^3^ Department of Internal Medicine I, Medical Faculty, Technische Universität Dresden, Dresden, Germany; ^4^ Early Clinical Trial Unit, Medical Faculty, Technische Universität Dresden, Dresden, Germany; ^5^ German Cancer Research Center (DKFZ), Heidelberg and German Cancer Consortium (DKTK) Partner Site, Dresden, Germany; ^6^ Department of Pharmaceutical Chemistry, University of Frankfurt, Frankfurt, Germany; ^7^ Max Planck Institute of Molecular Cell Biology and Genetics, Dresden, Germany; ^8^ National Center for Tumor Diseases (NCT), University Hospital Carl Gustav Carus, Technische Universität Dresden, Dresden, Germany; ^9^ Current address: Heinrich Pette Institute, Leibniz Institute for Experimental Virology, Hamburg, Germany

**Keywords:** AML, personalized medicine, UCN-01, STK3, mitosis

## Abstract

Acute myeloid leukemia (AML) is characterized by uncontrolled proliferation and accumulation of immature myeloblasts, which impair normal hematopoiesis. While this definition categorizes the disease into a distinctive group, the large number of different genetic and epigenetic alterations actually suggests that AML is not a single disease, but a plethora of malignancies. Still, most AML patients are not treated with targeted medication but rather by uniform approaches such as chemotherapy. The identification of novel treatment options likely requires the identification of cancer cell vulnerabilities that take into account the different genetic and epigenetic make-up of the individual tumors. Here we show that STK3 depletion by knock-down, knock-out or chemical inhibition results in apoptotic cells death in some but not all AML cell lines and primary cells tested. This effect is mediated by a premature activation of cyclin dependent kinase 1 (CDK1) in presence of elevated cyclin B1 levels. The anti-leukemic effects seen in both bulk and progenitor AML cells suggests that STK3 might be a promising target in a subset of AML patients.

## INTRODUCTION

Despite improvements through anthracycline dose escalation [1] and optimization of stem cell transplantation [2], AML remains a disease with dismal prognosis [3–5]. Since AML is a complex disease with diverse genetic background and considerable heterogeneity between patients [6–8], novel personalized forms of treatment focusing on individual vulnerabilities of a given leukemia may be necessary to improve the prognosis of AML patients.

Large-scale loss-of-function screens are useful to identify specific cancer cell vulnerabilities and several such screens have indeed identified novel and potent AML target genes [9–13]. From an RNAi screen performed in primary AML cells we have recently reported that STK3 knock-down significantly impaired cell growth in AML cells isolated from a patient [9].

STK3 (Serine/Threonine Kinase 3, also known as MST2) is one of the central kinases in the Hippo signaling pathway controlling cell proliferation, apoptosis and organ size [14–16]. Furthermore, it has been shown that STK3 suppresses tumorigenesis through inactivation of the YAP oncogene in several cancer type such as liver cancer [17] and lung cancer [18]. On the other hand, there are other studies suggesting that STK3 can mediate survival of cancerous cells including multiple myeloma and glioblastoma cells [19, 20]. Hence, the role of STK3 in tumorigenesis is ambiguous.

In the present study, we show that STK3 is required for cell growth and survival in a panel of established AML cell lines as well as in a number of primary AML samples. Interestingly, this effect seems to be independent of the Hippo signaling pathway. Rather, we see that STK3 depletion leads to cell death exclusively in the sensitive AML cells by modulating CDK1 activity and cyclin B1 levels, indicating that STK3 plays a direct or indirect role in cell cycle regulation in these cells. We furthermore identify the staurosporine derivative UCN-01 as a potent STK3 inhibitor and show that UCN-01 induced cell death predominantly results from STK3 inhibition in the tested AML cells. Our results suggest that STK3 is a promising drug target in selected AML cells that respond to STK3 inhibition due to their increased cyclin B1 levels and activated CDK1. Therefore, STK3 inhibition leads to a cell context dependent phenotype that could be exploited in personalized treatments of AML patients.

## RESULTS

### STK3 knock-down reduces cell proliferation in some AML cell lines

**Figure 1:**
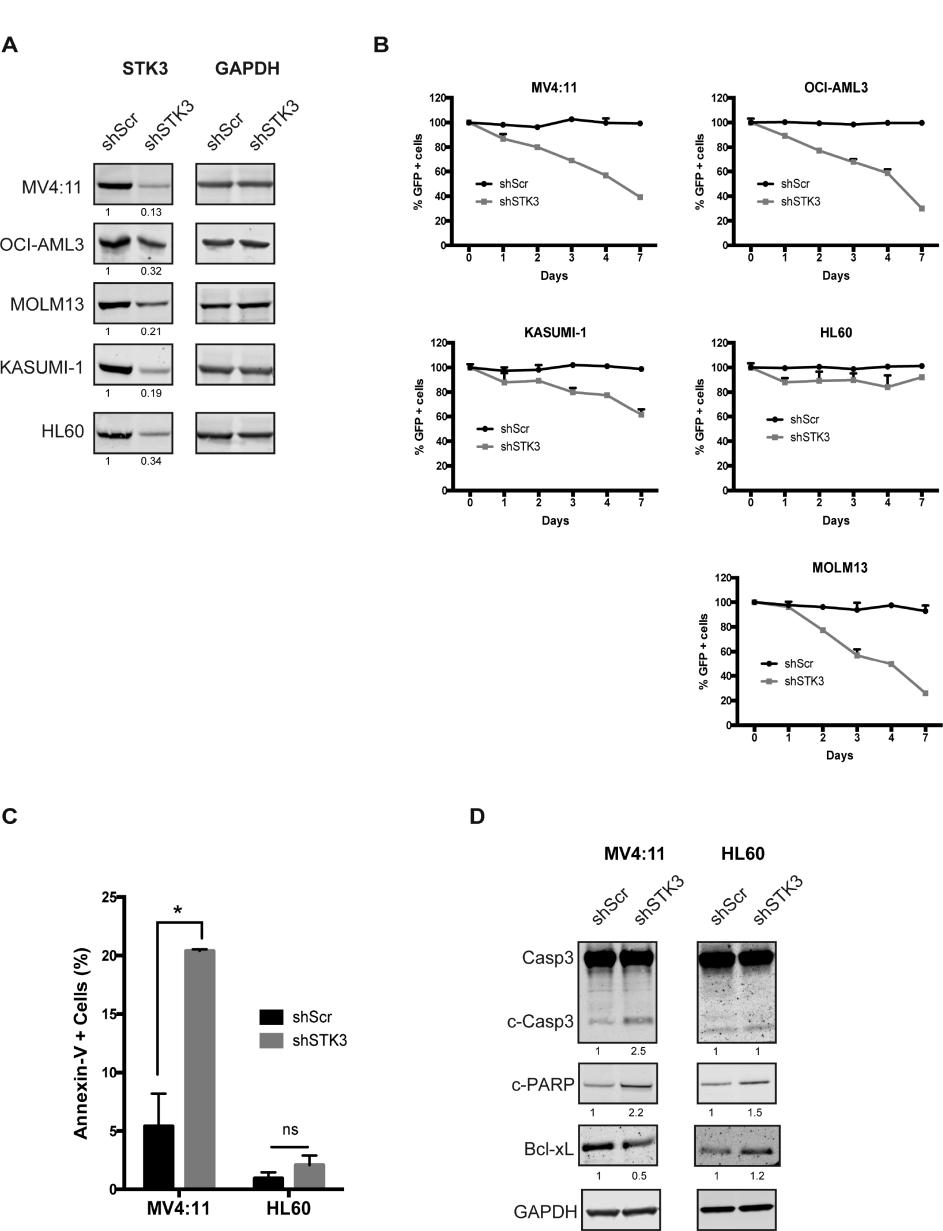
Effects of STK3 knock-down on AML cell lines (**A**) Knock-down levels of STK3 protein 3 days post infection are presented for each cell line. The quantitative values corresponding to each band after normalization to a loading control (GAPDH) are given below the western blot image. shScr, shRNA scrambled negative control. shSTK3, shRNA targeting STK3. (**B**) Indicated AML cell lines transduced with either GFP-tagged STK3 shRNA (shSTK3) or GFP-tagged non-target control shRNA (shScr) vectors. Percentage of GFP positive cells was checked by flow cytometry. GFP percentages normalized to day 2 post infection are presented. Changes in the abundance of GFP positive cells are shown over time. (**C**) STK3 knock-down induces apoptosis in MV4:11 cells. Quantitative analyses of Annexin V-positive cells after treatment with STK3 shRNA (shSTK3) or control shRNA (shSrc) in indicated cell lines are shown. Data are presented as mean ± SD. Significance was assessed by means of Student *t* test; ^*^P < 0.05 (**D**) Expression of apoptosis related proteins after STK3 knock-down examined by western blot assay in MV4:11 and HL60 cells at day 3 post infection is shown. The quantitative values normalized to GAPDH and the control shRNA are given below the western blot images.

In a genome-wide shRNA screen in cells isolated directly from an AML patient we had previously found that STK3 knock-down leads to decreased cell proliferation in bulk leukemic cells and might therefore be an interesting therapeutic target in this life-threatening disease. To test whether STK3 is required for proliferation in other AML cells, we depleted STK3 with a validated shRNA [9] in several established AML cell lines. To exclude the possibility of inefficient STK3 knock-down in different cell lines, we first analyzed STK3 protein levels by western blot analysis in shRNA transduced cells. Efficient knock-down was observed in all tested cell lines (Figure 1A), further validating the efficacy of the employed silencing trigger. We then investigated potential growth defects after STK3 depletion in these cells. After transducing cells with lentivirally expressed GFP in conjunction with shRNAs, we followed the percentage of GFP positive cells by flow cytometry in order to assess the effects resulting from STK3 knock-down. For a number of tested cell lines, STK3 knock-down resulted in a marked reduction in the percentage of GFP positive cells over time, as compared to control shRNA, indicating that these cell lines appear to be sensitive to STK3 knock-down. Interestingly, STK3 knock-down in HL60 cells did not show this phenotype with the percentage of GFP positive cells remaining stable and comparable to cells transduced with the control shRNA (Figure 1B). To investigate why GFP levels vanish in sensitive cells, we depleted STK3 in MV4:11 and HL60 cells and measured apoptosis rates over time. No increase in apoptotic cells was observed after STK3 depletion in HL60 cells. In contrast, a significant increase in Annexin V positive cells was apparent after STK3 knock-down in MV4:11 cells (Figure 1C), demonstrating that STK3 expression was required for cell survival in these cells. Further analyzes of the levels of pro-apoptotic (cleaved Caspase3 and cleaved PARP) and anti-apoptotic (Bcl-xL) proteins by western blot (Figure 1D) confirmed that knock-down of STK3 results in an apoptosis induced cell death in MV4:11 cells but not in HL60 cells.

### CRISPR-Cas9 mediated STK3 knock-out in MV4:11 cells phenocopies RNAi results

**Figure 2:**
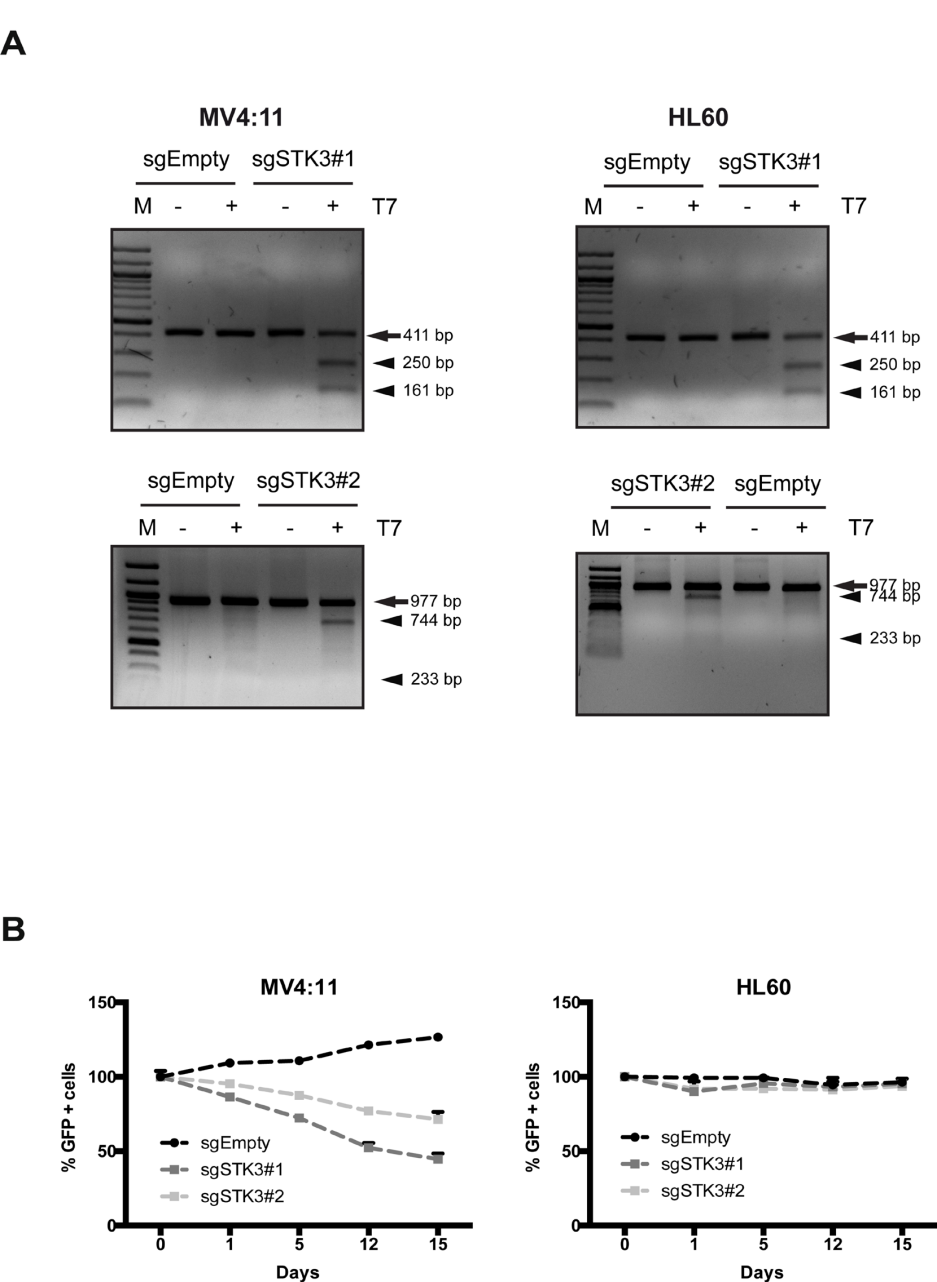
STK3 knock-out using CRISPR-Cas9 phenocopies RNAi results (**A**) MV4:11 and HL60 cells were transduced with vectors containing two different gRNAs targeting STK3 (sgSTK3#1 and sgSTK3#2) or an empty vector (sgEmpty, negative control). Genomic PCR prepared from cells with indicated treatments in an T7E1 assay are shown. Arrows indicate the size of wild-type PCR products, arrowheads indicate the expected cleavage products of the T7E1 assays. (**B**) Effects of STK3 knock-out in MV4:11 and HL60 cells. Changes in the percentage of GFP+ cells are presented after normalization. GFP percentage was normalized to day 2 post infection and presented as day 0. Data are presented as mean ± SD.

To exclude a possible shRNA-mediated off-target effect of the observed RNAi phenotype, we investigated the effect of CRISPR-Cas9 mediated STK3 knock-out in MV4:11 and HL60 cells. We designed two STK3-specific gRNAs and tested their cleavage efficiency when expressed together with Cas9. Delivery of gRNAs and a Cas9-GFP fusion via lentiviral-transduction resulted in the expected cleavage products exclusively in cells treated with the STK3 gRNA, indicating the presence of indels in the target sequence (Figure 2A). To investigate a possible phenotype of STK3 inactivation, we again followed the percentage of GFP positive cells over time in unsorted cell populations after transduction. In agreement with the RNAi results, a decrease of GFP positive cells was observed in MV4:11 cells, but not in HL60 cells (Figure 2B). Hence, these results confirm that inactivation of STK3 leads to a proliferative defect in a cell line-specific manner.

### STK3 depletion exerts anti-leukemic effects in primary AML cells

**Figure 3:**
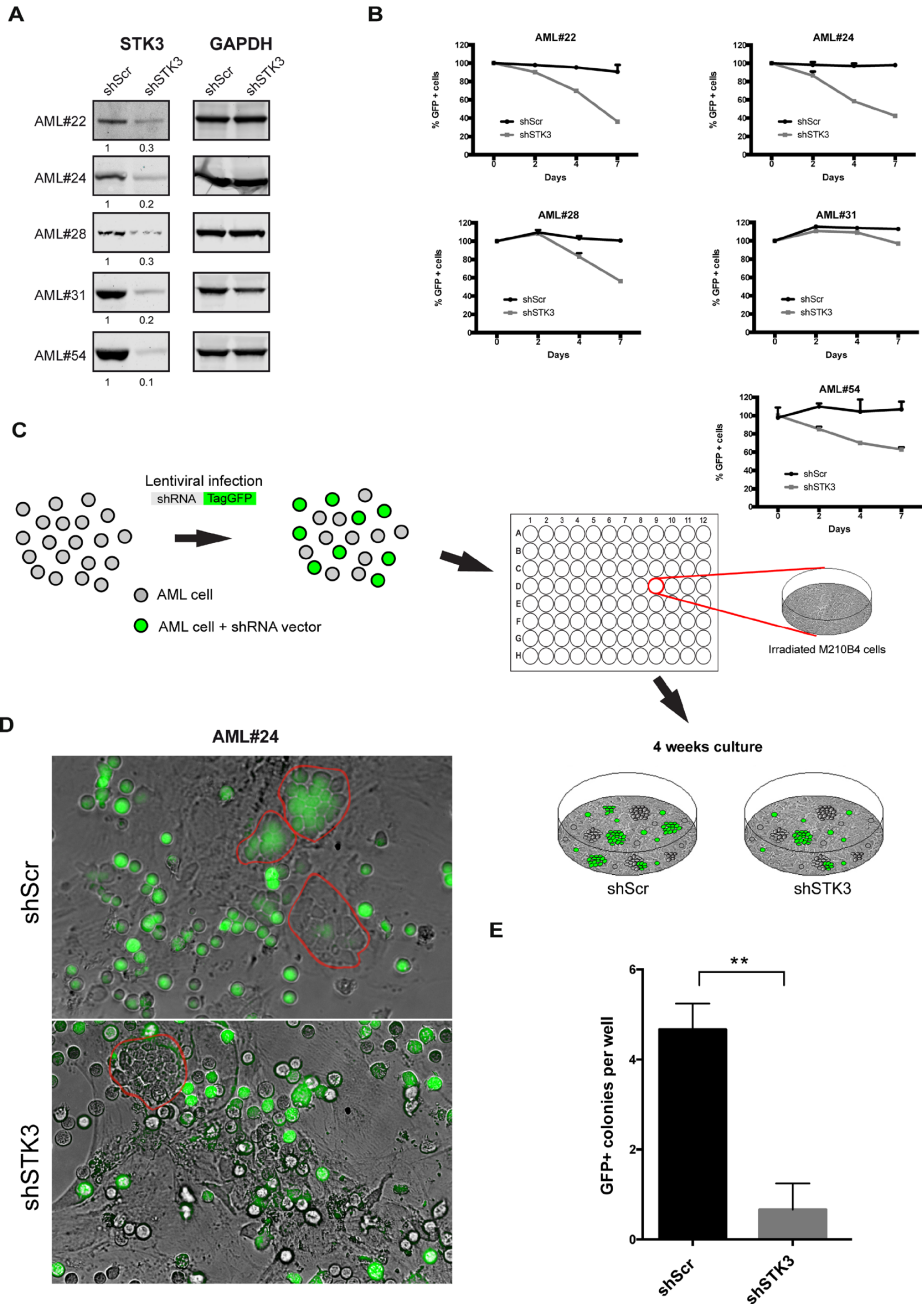
Effects of STK3 knock-down on AML blast- and progenitor- cells (**A**) Knock-down of STK3 in primary AML cells. Western blots show protein levels 4 days post infection with indicated shRNAs. The quantitative values corresponding to each band after normalization to loading control (GAPDH) are given below the western blot images. (**B**) Phenotypes of STK3 knock-down in primary AMLs. Cells transduced with either GFP-tagged STK3 shRNA (shSTK3) or GFP-tagged non-target control shRNA (shScr) vectors are shown. GFP percentage was normalized to day 2 post infection and presented as day 0. Changes in the GFP percentages are shown at indicated time points. (**C**) Scheme of the experimental design of LTC-IC. Important steps are indicated by arrows. (**D**) Representative microscopic images of leukemic progenitors of AML#24 transduced with indicated shRNAs in LTC-IC assays. GFP positive and negative cobble stones are highlighted by red circles. (**E**) Quantitative analysis of experiment from panel (D). GFP+ colonies were counted in each well (*n* = 3 per each shRNA vector). Data are presented as mean ± SD. Significance was assessed by means of Student *t* test; ^**^P < 0.01.

To investigate whether primary AML patient samples also show differential sensitivity to STK3 depletion, we tested cells from 5 different patients. We first confirmed efficient STK3 knock-down on protein level in all tested samples (Figure 3A). To measure proliferative effects upon STK3 knock-down we again transduced the cells with lentiviral vectors expressing control-, or STK3-targeting shRNAs and followed the percentage of GFP positive cells over time. Similar to the results obtained in cell lines, STK3 knock-down showed significant reduction in the percentage of GFP positive cells compared to cells expressing control shRNA, in some but not in all AML patient samples (Figure 3B). Moreover, CD34+ HSPCs of 2 healthy donors appeared to be largely resistant to shRNA-mediated STK3 depletion compared to sensitive AML patient samples (Supplementary Figure 1). Hence, like established cell lines, some primary AML cells are sensitive to STK3 depletion while others show no growth defect.

To investigate a possible effect of STK3 knock-down on more immature AML progenitor cells, we performed RNAi in combination with long-term culture-initiating colony (LTC-IC) assays (Figure 3C). STK3 or control shRNA- transduced cells were co-cultured for four weeks on irradiated M2-10B4 feeder cells before analyzing the formation of cobble-stone areas, which are thought to represent clonal growth of progenitors with stem cell like characteristics [21]. After infection with the control shRNA vector, the wells contained an equal mixture of GFP positive and negative colonies, with on average 8–10 cobble-stones forming per well. In contrast, AML cells expressing the STK3 targeting shRNA showed a much-reduced number of GFP positive cobble-stones (0–1 cobble-stone per well), whereas GFP-negative cobble-stone formation was unaffected (Figure 3D, 3E). This data indicates that not only bulk AML cells but also more immature leukemic cells require STK3 for survival.

### UCN-01 induces cell death in AML cells sensitive to STK3 depletion

**Figure 4:**
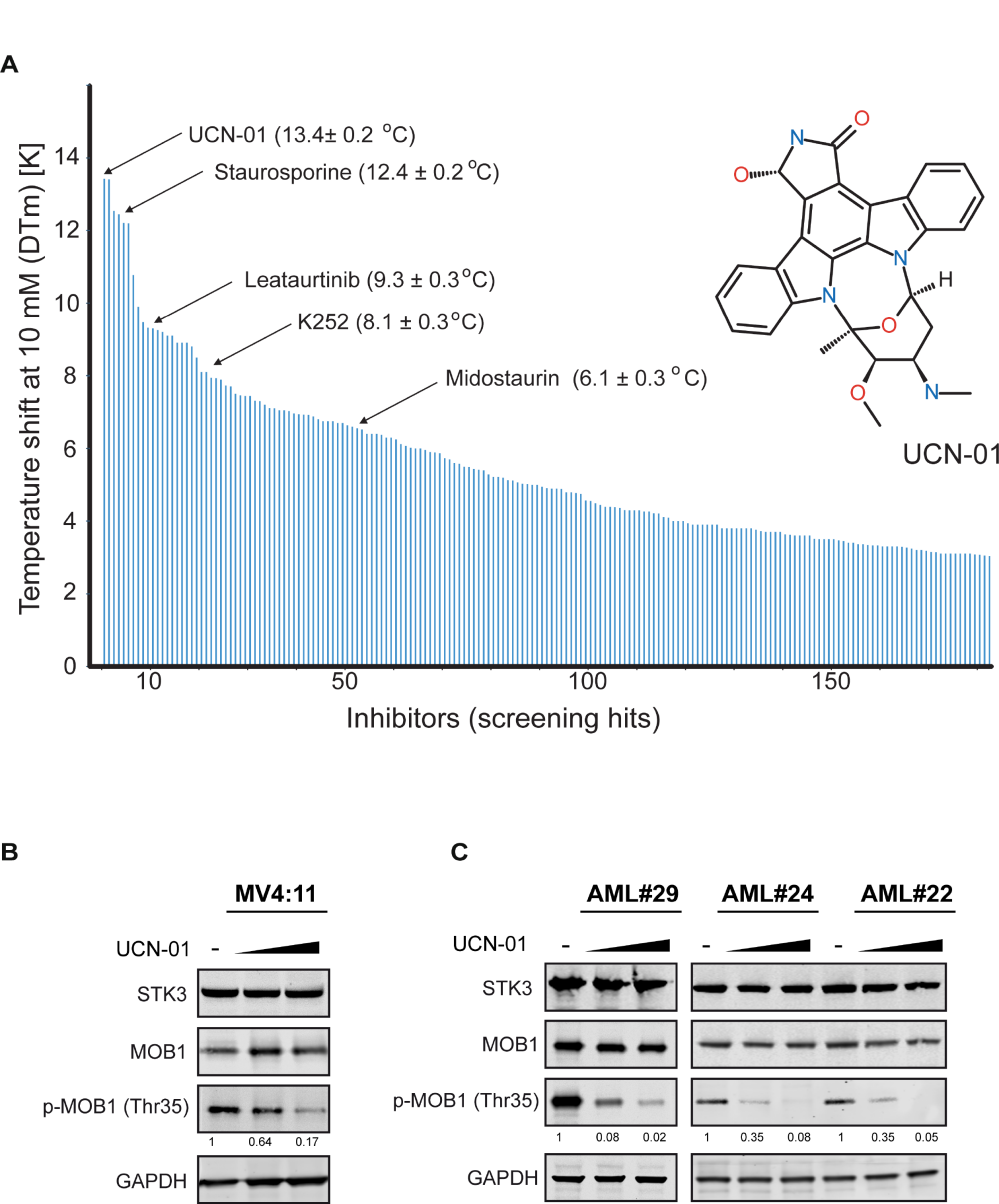
Multikinase inhibitor UCN-01 as a STK3 inhibitor (**A**) *In vitro* compound screen identified UCN-01 as a strong STK3 binding partner. Each line represents a compound with a temperature shift >3° C. Staurosporine-derivatives with their temperature shift differences are highlighted by their names. The structural formula of UCN-01 is shown on the right. (**B**) Treatment of MV4:11 cells with UCN-01 affects MOB1 phosphorylation. Increasing concentrations of UCN-01 (150 nM and 500 nM) were tested for 4 h and MOB1 phosphorylation was assessed by a western blot assay. Quantification of the p-MOB1 band intensities, normalized to GAPDH, are shown. (**C**) Treatment of primary AML cells with UCN-01 affects MOB1 phosphorylation. Increasing concentrations of UCN-01 (150 nM and 500 nM) were tested for 18 h and MOB1 phosphorylation was assessed by a western blot assay. Quantification of the p-MOB1 band intensities, normalized to GAPDH, are shown.

In search for a chemical inhibitor targeting STK3 kinase activity, we screened a chemical compound library of about 3000 commercially available kinase inhibitors against the STK3 kinase domain using temperature shift (ΔT_m_) assays [22]. Screening this library, we identified 184 inhibitors with shifts larger than 3 degrees. The strongest hit was the staurosporine derivative UCN-01 along with other derivatives of this scaffold (Figure 4A). UCN-01 has narrow kinase selectivity compared to the broad spectrum inhibitor staurosporine but it has been described to inhibit diverse kinases [23–26]. However, inhibition of STK3 has not been reported so far. To investigate whether UCN-01 inhibits STK3 kinase activity in cells, we treated MV4:11 cells with increasing concentrations of UCN-01 and subsequently analyzed phospho-MOB1 levels, a validated direct target of STK3 [27, 28]. Without changes in either total STK3 protein or total MOB1 protein expression, phosphorylated MOB1 levels diminished in a dose dependent manner (Figure 4B). Similar results were also obtained in several primary AML cells (Figure 4C), demonstrating that UCN-1 treatment reduces STK3 activity in a dose dependent manner.

**Figure 5:**
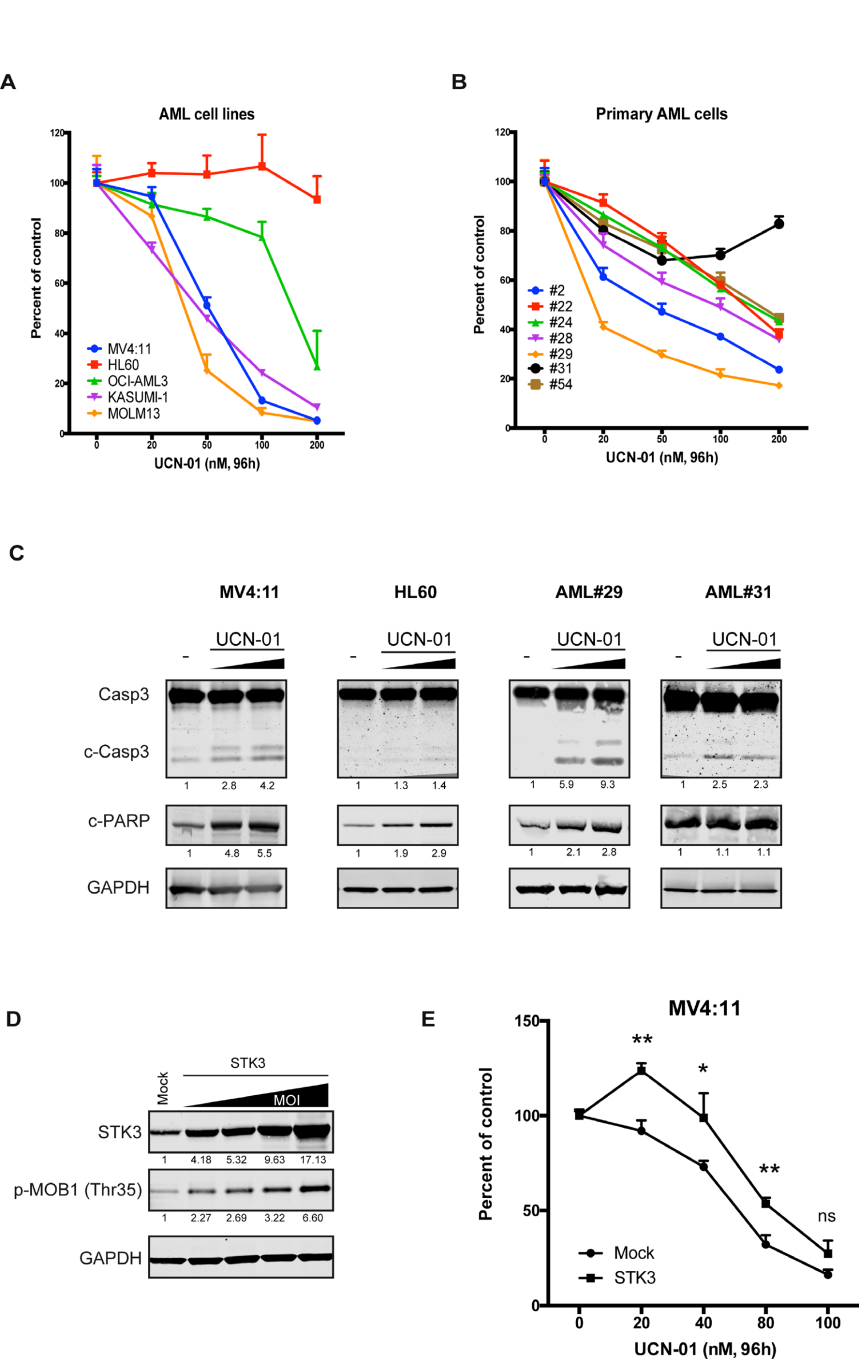
Inhibition of STK3 by UCN-01 phenocopies STK3 knock-down data in AML cells (**A**) Dose-escalation effect of UCN-01 on selected AML cell lines. Cell survival after treatment with increasing concentrations of UCN-01 for 96 h in indicated AML cell lines is shown. (**B**) Dose-escalation effect of UCN-01 on selected primary AML cells. Cell survival after treatment with increasing concentrations of UCN-01 for 96 h in primary AML cells is shown. The results are presented as percentages of the control condition (only DMSO as a vehicle) for each sample. Data are presented as mean ± SD in A and B panel. (**C**) Expression of apoptosis related proteins after increasing concentration of UCN-01 (150 nM and 500 nM) examined by western blot assay in AML cell lines (MV4:11 and HL60) after 4 h and primary AML cells (AML#29 and AML#31) after 18 h are shown. The quantitative values normalized to GAPDH and the control are given below the western blot images. (**D**) Expression of exogenous STK3 increases phospho-MOB1 (p-MOB1) levels in MV4:11 cells. Increasing multiplicity of infection (MOI) resulted in increased phosphorylation of MOB1. Quantifications of bands are shown below the blot image as a percentage of mock control. (**E**) Over-expression of STK3 affects UCN-01 induced cell death compared to cells transduced with mock vector. Data are presented as mean ± SD. Significance was assessed by means of Student *t* test; ^*^P < 0.05, ^**^P < 0.01.

To test if UCN-01 treatment affects AML cell line growth, we incubated cells with increasing doses of UCN-01 and monitored proliferation rates. Remarkably, the results revealed a strong correlation between UCN-01 sensitivity and STK3 knock-down response in the tested lines (Figure 5A). Furthermore, this correlation also extended to primary AML samples (Figure 5B). In particular, HL60 and AML#31 cells were resistant to UCN-1 treatment, whereas other samples showed sensitivity to inhibitor treatment in a dose dependent manner. Moreover, further analyzes of the levels of pro-apoptotic (cleaved Caspase3 and cleaved PARP) proteins by western blot confirmed that UCN-01 induces apoptosis in a dose dependent manner primarily in MV4:11 and AML#29 cells but not in HL60 and AML#31 cells (Figure 5C). To validate a causal link between the UCN-1 induced proliferative effects via STK3 inhibition, we performed STK3 over-expression studies in combination with UCN-1 treatment. We reasoned that over-expression of STK3 should make the cells more resistant to UCN-01 treatment. We first confirmed that over-expression of STK3 gave rise to a functional form of the STK3 kinase based on the increased phosphorylation levels of the downstream substrate MOB1 (Figure 5D). We then applied UCN-01 to cells at increasing concentrations and compared the growth rates of wild-type and STK3 over-expressing cells. We observed that MV4:11 cells over-expressing STK3 were more resistant to drug exposure (Figure 5E), further supporting that UCN-1 inhibits STK3 and that this inhibition leads to cell death in sensitive AML cells.

We conclude that despite its activity on other kinases, UCN-1 treatment phenocopies the genetic depletion experiments in AML cells, suggesting that UCN-1 induced cell death in these cells is primarily a result of STK3 inhibition.

### The Hippo pathway co-activator YAP is not involved in STK3 inhibition mediated cell death

To gain insight into the molecular mechanism of STK3 depletion/inhibition caused cell death in leukemic cells, we decided to investigate the Hippo signaling cascade, of which STK3 is a central kinase [29]. YAP is the central downstream effector of the canonical Hippo pathway [30–32] and was therefore investigated in AML cells. To examine the base-line expression of YAP in the utilized AML cell lines we performed qRT-PCR and Western blot analyses. Surprisingly, YAP was undetectable in most of the cell lines (Supplementary Figure 2A). Likewise, expression of YAP was barely detectable on mRNA level and undetectable on protein level in the tested primary AML samples (Supplementary Figure 2B). More importantly, YAP levels did not change and were therefore also not detectable in most cells after UCN-01 treatment, STK3 knock-down, or STK3 over-expression (Supplementary Figure 2C, 2D). Hence, alteration of the canonical Hippo pathway is unlikely the cause of cell death after STK3 inhibition in sensitive AML cells.

### STK3 modulates CDK1/Cyclin B1 activity in a subset of AMLs

**Figure 6:**
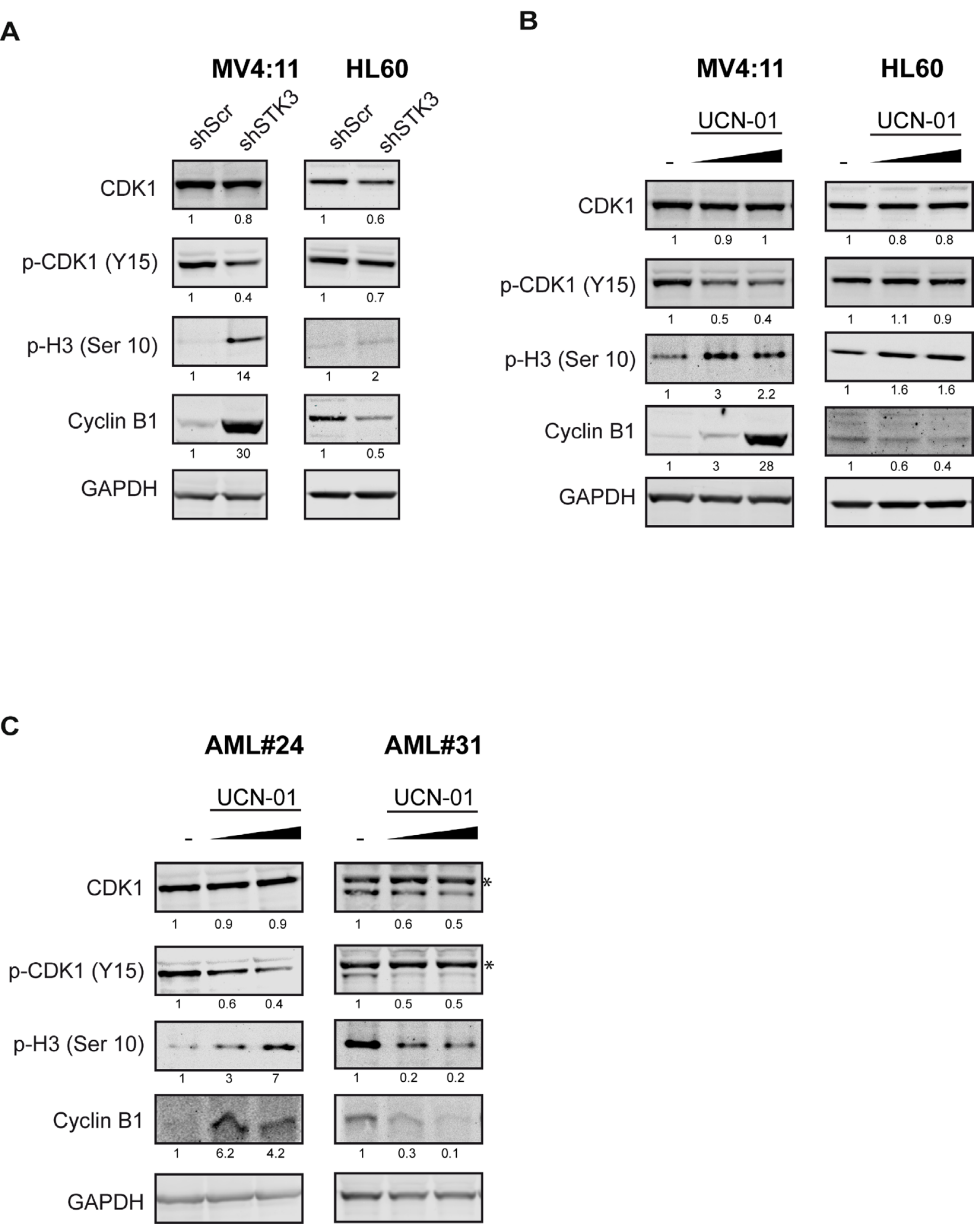
STK3 depletion or inhibition leads to growth inhibition through modulation of CDK1/cyclin B1 activity in sensitive AML cells (**A**) Representative western blot analysis of indicated mitosis related proteins and their respective phosphorylated versions (p-) after STK3 knock-down in AML cell lines (MV4:11 and HL60, respectively). (**B**) Representative western blot analysis of indicated mitosis related proteins and their respective phosphorylated versions (p-) after 4 h treatment with UCN-01 in indicated AML cell lines. (**C**) Representative western blot analysis of indicated mitosis related proteins and their respective phosphorylated versions (p-) after 18 h treatment with UCN-01 in indicated primary AML cells (AML#24 and AML#31, respectively). Quantifications of bands are shown below the blot image as a percentage of scramble control or vehicle (DMSO) treated control. ^*^ indicates unspecific bands.

To further analyze the molecular mechanisms by which STK3 depletion induces apoptosis, we performed an unbiased phospho-proteomic experiment, in order to uncover proteins that are differentially phosphorylated. In this experiment, we compared the total phospho-proteome of cells transduced with STK3 shRNA or a non-targeting shRNA. Analysis of the data revealed a panel of differentially phosphorylated proteins, unmasking direct and indirect STK3 targets (Supplementary Table 1). Interestingly, Gene Ontology Enrichment Analysis (GO) revealed that one of the most affected GO processes after STK3 knock-down was cell cycle, implicating STK3 kinase activity in this pathway (Supplementary Figure 3). Indeed, STK3 has been previously shown to regulate mitotic progression [33–35]. We therefore decided to investigate whether the cell line-specific phenotype could be explained by a differential influence on mitotic cell cycle proteins. Strikingly, depletion of STK3 resulted in very distinct modulation of cell cycle proteins in sensitive versus resistant cell lines. While in sensitive MV4:11 cells STK3 knock-down resulted in reduced phosphorylation of CDK1 at tyrosine 15 (the inhibitory phosphorylation site of CDK1) and increased phosphorylation of histone H3 at Serine 10, little to no changes were detected in resistant HL60 cells. This difference in phosphorylation was accompanied by a prominent difference in abundance of cyclin B1, which is a crucial protein for mitotic progression. While in HL60 cells cyclin B1 level dropped after STK3 knock-down, a marked increase of this protein was observed in MV4:11 cells (Figure 6A). Reduction of CDK1 phosphorylation and strongly increased cyclin B1 levels were also solely detected in MV4:11 cells upon UCN-01 treatment in a dose-dependent manner (Figure 6B), further supporting that STK3 depletion has an impact on cell cycle proteins in a cell line-specific manner.

**Figure 7:**
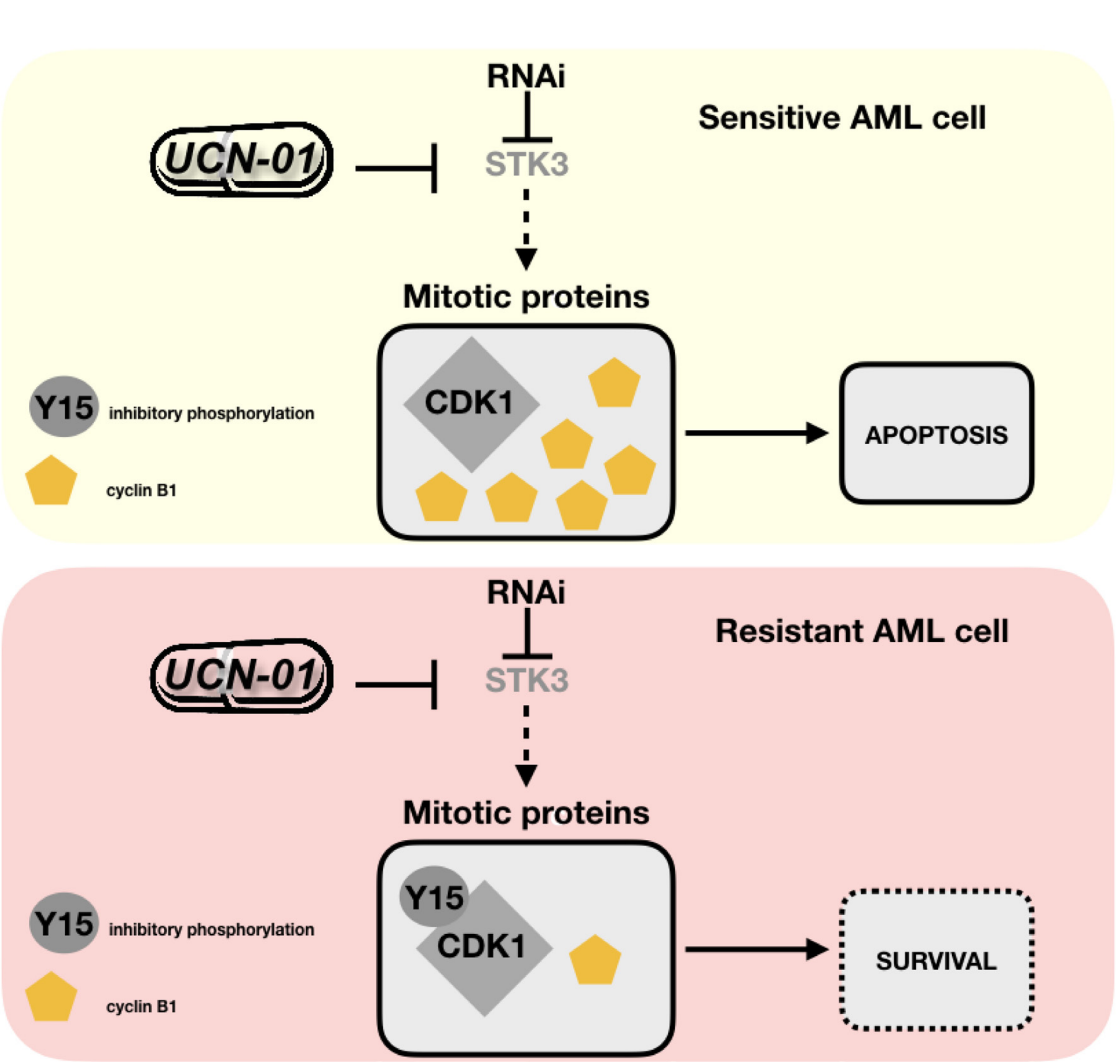
Model to explain STK3 depletion sensitivity versus resistance in AML cells Sensitive AML cells react to STK3 inhibition/depletion by diminished inhibitory phosphorylation (Y15) of CDK1 and with accumulation of cyclin B1. This aberrant activation of cell cycle proteins leads to apoptosis. Resistant AML cells do not show this effect and survive STK3 inhibition/depletion.

We finally tested if this effect is also seen in a pair of STK3-sensitive and –resistant primary AML samples. Indeed, we observed that the inhibitory phosphorylation site of CDK1 (p-Y15) decreased in the presence of elevated cyclin B1 levels in a dose dependent manner exclusively in the sensitive AML patient sample (AML#24), but not in the resistant patient sample (AML#31, Figure 6C). In line with AML cell line results, our results support a model where STK3 inhibition leads to aberrant activation of CDK1/cyclin B1 in a subset of AML cell lines and primary patient samples, which triggers cell death specifically in these cells (Figure 7).

## DISCUSSION

Advances in DNA sequencing techniques are providing an unprecedented view of the complex genetic heterogeneity within individual tumors. This genetic heterogeneity is accompanied by phenotypic heterogeneity, where perturbations lead to differential effects in different tumor cells. These circumstances make tumor research more complex, but it also offers new possibilities for innovative treatments and constitutes efforts in precision oncology.

In this study, we unmasked the vulnerability of some AML cells to depletion of serine/threonine-protein kinase 3, a central kinase of the Hippo signaling pathway [15, 32, 36–39]. Hippo signaling plays in important role in tumor development and maintenance [40]. However, in this case, altered Hippo signaling does not seem to account for the growth defects in sensitive AML cells. Instead, we show that this dependency is linked to a cell line-contingent response of the cell cycle proteins CDK1 and cyclin B1 to STK3 depletion. CDK1 is one of the major protein kinases for promoting mitosis. Its activity in mitosis is mainly controlled by direct phosphorylation/de-phosphorylation of substrates and indirectly through its cofactor cyclin B1 [41–43]. Of note, CDK1 improper activation together with cyclin B1 accumulation in cells has been linked to induction of mitotic catastrophe and cell death [44–48].

It is therefore likely that STK3-mediated depletion leads to apoptosis via accumulation of CDK1/cyclin B1 in sensitive AML cells. Why some cells react in this way while others do not is currently not clear. It is possible that STK3 indirectly influences the inhibitory phosphorylation site of CDK1 (p-Y15) in the sensitive cells, while a parallel pathway for this phosphorylation event is operating in the resistant cells. Additional work is required to investigate this possibility in the future. Nevertheless, the identification of a molecular marker that distinguishes sensitive from resistant AML cells could be used in a future precision oncology driven approach to find specific vulnerabilities in patient samples.

Our work also discovered UCN-01, an inhibitor that has been demonstrated to inhibit CHK1, AKT and PDK1 [23, 49, 50], as a potent inhibitor of STK3. The ability of UCN-01 to inhibit several kinases makes it an attractive drug candidate and it has indeed been evaluated in several phase I-II clinical trials alone or in combination with other genotoxic drugs [51–54], including a phase I trial in AML [55]. Unfortunately, none of these trials have generated strong enough data to support further development of this drug candidate for cancer treatment. However, most of these trials have focused on the ability of UCN-01 to inhibit CHK1, or AKT. We suggest that it might be useful to reevaluate the obtained data with a view of UCN-01 as an STK3 inhibitor. We further note that Midostaurin, a recently approved drug for the treatment of adult patients with newly diagnosed AML who are FLT3 mutation-positive [56], was among the strong STK3 binders. It might therefore be interesting to investigate whether the observed anti-leukemic activity of the drug is influenced by its ability to inhibit STK3 and thereby aberrantly activate CDK1/cyclin B1.

Because UCN-01 is also inhibiting other kinases, a more specific STK3 inhibitor would be very helpful for mechanistic studies on the role of STK3 in signaling and cancer. Indeed, a compound with reversible and selective STK3 inhibitory characteristics has recently been described [57]. Obtaining a mechanistic understanding of the differential effect on STK3 depletion in individual AML samples would advance our understanding of the development of selective vulnerabilities in cancer and provide novel opportunities for precision oncology. Providing a rationale for further clinical evaluations might make STK3 a novel therapeutic target for some AML patients in order to develop better treatment options.

## MATERIALS AND METHODS

### Inhibitors

UCN-01 was purchased from Sigma-Aldrich. Stock solution was prepared as 5 mM in DMSO. The stock solutions were kept at −20° C until the next usage. For further dilutions cell culture medium was used.

### Cell lines and primary cells

MV4:11, MOLM13, OCI-AML3, KASUMI-1 and HL60 cell lines were kindly provided by Prof. Dr. Martin Bornhäuser. M2-10B4 murine bone marrow stromal cells (MBSCs) were purchased from the American Type Culture Collection (ATCC). Cell lines were cultured in RPMI 1640 medium (MV4:11, MOLM13, KASUMI-1 and HL60) or Alpha-MEM medium (OCI-AML3) supplemented with 10% heat-inactivated fetal bovine serum (GIBCO), 1% L-glutamine and penicillin-streptomycin in a humid environment of 5% CO_2_ at 37°C. All AML cell lines used in this study are described in Supplementary Table 2A.

Primary cells from patients were obtained from bone marrow samples or peripheral blood of leukemic patients. Hematopoietic stem/progenitor cells (HSPCs) were harvested from leukapheresis of healthy donors and selected for CD34 (MACS MicroBeads; Miltenyi Biotec). All primary cells underwent controlled-rate freezing and were subsequently stored in the vapor phase of liquid nitrogen. Freshly thawed B- and T-lymphocyte-depleted (MACS MicroBeads; Miltenyi Biotec) leukemic or normal HSPCs were used for all experiments. All primary cells were grown in StemSpan SFEM with 2% fetal calf serum (both STEMCELL Technologies) supplemented with 10 ng/ml of rhIL3, rhTPO, rhFLT3-ligand, and rhSCF (all R&D Systems). The purity was <0.5% lymphocytes for leukemic cell populations and >95% CD34+ for normal HSPCs as assessed by flow cytometry. All primary cells were obtained under protocols approved by the local institutional review board. Moreover, all patients were included in the AML registry of the Study Alliance Leukemia and agreed to the use of their samples for research. This prospective observational trial has been registered with www.Clinicaltrials.gov (NCT03188874). Supplementary Table 2B contains the clinical features of the AML patient samples used throughout the study.

### Plasmids

shRNA sequences were cloned and expressed from pRSI12_U6_shRNA_Ubic_TagGFP_2A_Puro (pRSI12) vector (Cellecta) after cutting with BbsI restriction enzyme (New England Biolabs). pRSI12 based shRNA vectors were used for knocking down STK3 throughout the study unless otherwise stated. For expression of Cas9 and STK3 sgRNAs from lentiviral plasmids pLCRISPR.EFS.GFP was used (Addgene plasmid # 57818). For over-expression of STK3, the coding sequence of full-length STK3 (isoform 1, ENST00000419617.6) was amplified from MV4:11 cDNA by PCR using the following two primers 5′-ATGGAGCAGCCGCCGG-3′ and 5′-TCAAAAGTTTTGCTGCCTTCTTTTC-3′. In the second PCR step, restriction sites (BamHI and Sal I) were added using the following two primers 5′-TTTGGATCCGAGGGCAGAGGAAGCCTTCTAACATGCGGTGACGTGGAGGAGAATCCCGGCCCTATGGAGCAGCCGCCGG-3′ and 5′-AAAGTCGACCAAGATGGCCATATGTAGCTTAGGAAATGACTGGTCCCAATTCAAAAGTTTTGCTGCCTTCTT-3′. The final PCR product was subcloned into pRSI12 vector in place of the Puro cassette. All constructs were confirmed by DNA sequencing. The sequence information can be found in Supplementary Table 3.

### Lentiviral infection of cells and cell growth assessment

Lentiviral particle production and infection were performed as previously published [58]. Briefly, cells were seeded in either 12 or 24 well plates coated with RetroNectin^**®**^ **(**Takara Clontech). Transduction was performed in the presence of protamine sulfate (final concentration 5 ug/mL, Sigma-Aldrich) and spin-infected for 1 h at 1,000 g and 37° C. Throughout the study we used an MOI between 0.3–1 in AML cells. Transduced cells were further cultured and analyzed by MACSQuant Analyzer (Miltenyi Biotec and FlowJO software) for GFP expression. Viable cells were discriminated from dead cells by DAPI exclusion. Reduction of the percentage of GFP-positive cells indicates that the infected cells expressing a particular shRNA have a growth disadvantage in comparison to the non-infected cells.

### Apoptosis

MV4:11 and HL60 cells were infected either with pLKO.1 STK3 shRNA vector or the pLKO.1 scramble control. At day 2 post infection cells were selected for with puromycin for an additional 2 days (2 ug/ml) to deplete uninfected cells. Two days later, apoptotic cells were detected using an Annexin V_FITC kit (Miltenyi Biotec) following the manufacturer’s instruction.

### CRISPR-Cas9 mediated STK3 knock-out

sgRNAs targeting SKT3 were designed using the algorithm developed by Sullender *et al.* [59]. On target effects of sgRNAs were evaluated by detecting indel formation with the T7 endonuclease I assay (T7EI) [60]. At day 3 post infection, cells were sorted for GFP expression using a BD FACSAria^™^ to isolate genomic DNA to perform T7EI assays. Unsorted MV4:11 and HL60 cells were analyzed over time for percentage of GFP positive cells using MACSQuant and FlowJO software. Viable cells were discriminated from dead cells by DAPI exclusion.

### Long term culture initiating cells assay (LTC-IC)

For LTC-IC assays, 1 × 10^3^ AML#24 cells per well were seeded in MyeloCult H5100 (STEMCELL Technologies) on 4 × 10^4^ irradiated MBSCs per well at day 1 post infection in a 96 well plate (3 well for each shRNA). To check initial GFP percentage of cells in shScr and shSTK3 population, half of the transduced cells were kept in parallel in the suspension culture for another 2 days. GFP levels were then recorded by flow cytometry (shScr 53%, shSTK3 55.3%). After 4 weeks, cobblestone areas were manually counted in each well. Cobblestone areas were defined as any area of >5 hematopoietic cells growing immediately adjacent to each other and having a rectangular shape. Images were taken using an EVOS FL Cell Imaging System (Life Technologies) with 20× objective.

### *In vitro* compound screen

The catalytic domain of STK3 has been expressed in bacteria as a HIS-tagged protein as described [61]. ΔT_m_ assays have been carried out at 10 µM compound concentration as described in Fedorov *et al.* [22].

### *In vitro* cell viability assay after drug treatment

For primary AML cells 1 × 10^5^ cells and for AML cell lines (MV4:11 and HL60) 5 × 10^4^ cells were seeded in 96-well culture plates with increasing concentrations of UCN-01 for 96 hours. Cell viability was determined by MACSQuant. 4’,6-diamidino-2-phenylindole (DAPI) was added to the cells at a final concentration of 40 ng/ml for dead/viable cell discrimination just before analysis. Data were presented as percent of only DMSO treated cells (control cells). DMSO concentrations were always less than 0.5% in the culture medium and equal in all drugs assays.

### Assessment of protein expression by western blot

After knock-down or knock-out of STK3 and after UCN-01 treatment, changes in protein expression was evaluated by Western blot. The cells were lysed in commercial cell lysis buffer (Cell Signaling Technology) containing Halt protease and phosphatase inhibitor cocktail (Thermo Scientific). Protein concentration was measured by 660 nM Protein Assay (Thermo Scientific) and equal amounts of total proteins were resolved on 4–12% SDS-PAGE gels (Life Technology) after denaturation in LDS loading buffer (Life Technology) at 70° C for 10 min. Proteins were transferred to nitrocellulose membranes (Amersham Protran). Membranes were blocked with either 5% BSA (for phosphorylated proteins) or 5% nonfat dry milk in PBS-T (0.1% [v/v] Tween-20, Serva) for 1 h at room temperature, and then incubated with primary antibodies in related blocking buffer at 4° C overnight. Primary antibodies against STK3 (ab52641), CDK1 (ab32384), MOB1 (#3863), T-35 MOB1 (#8699), cleaved PARP1 (#9541), Bcl-xL (#2762), Y-15 CDK1 (#4539), Ser-10 Histone3 (#3377), Cyclin B1 (554177), Caspase3 active and cleaved (NB100-56708), GAPDH (NB-300-221),) were purchased from Abcam, Cell Signaling Technology, BD and Novus, respectively. The membranes were washed twice for 10 min each in PBS-T before probing with donkey anti-mouse/rabbit/goat IR-Dye 670 or 800cw labeled secondary antibody in blocking buffer for 1 h at room temperature. After washing twice for 10 min in PBS-T, membranes were placed in PBS. Membranes were imaged using a LiCor Odyssey scanner. Boxes were manually placed around each band of interest, which returned near-infrared fluorescent values of raw intensity with intra-lane background subtracted using the Image Studio Lite program (Version4, LiCor).

### Quantitative real-time RT-PCR

To assess mRNA expression of the Hippo-pathway gene YAP real-time quantitative reverse transcriptase PCR (qRT-PCR) was conducted. Isolation of mRNA was performed according to RNeasy Mini Kit protocol (Qiagen) with on column DNaseI digest (Qiagen). cDNA was synthesized according to the SuperScript III Reverse Transcriptase protocol (Invitrogen GmbH) by using 1ug total RNA for each reaction. Quantitative PCR was performed using the Absolute qPCR 2× SYBR Green Kit (Thermo Scientific) following the manufacture protocol on C1000 Touch^™^ Thermal Cycler (Bio-Rad). Primers used for YAP gene; forward 5′-CCTTCTTCAAGCCGCCGGAG-3′ and reverse 5′-CAGTGTCCCAGGAGAAACAGC-3′, for GAPDH gene; forward 5′-GCACCGTCAAGGCTGAGAAC-3′ and reverse 5′-AGGGATCTCGCTCCTGGAA-3. Using the 2-ΔΔCt method, mRNA expression results were first normalized against GAPDH as internal control and then normalized to Hela expression results as a positive control.

### Mass spectrometry based phospho-proteomics analysis

MV4:11 cells were infected either with pRSI12 STK3 shRNA vector or pRSI12 scramble control. At day 2 post infection cells were selected with puromycin for an additional 2 days (2 ug/ml) to deplete uninfected cells. Cells were propagated in culture for one additional day. After snap freezing, the cells were stored at –80° C and later processed for mass spectrometry analysis. Cell pellets were dissolved in 6 M Guanidium Hydrochloride (GnHCL) in 100 mM HEPES pH 8.5 containing 5 mM TCEP and 10 mM CAA. Lysates were diluted to 2 M GnHCL for LysC digestion (1 ug for 100 ug lysate) and further to 1M for trypsin digestion (1ug for 100 ug lysate). Digested peptides were desalted using a C18 Sep-Pak cartridge (Waters) and eluted with 50% acetonitrile. 450 ug of purified peptides were TMT labelled per channel according to the manufacture´s instruction with some modifications [62]. TMT labelling was quenched using 5% hydroxylamine and combined for further high pH reversed phase fractionation and phospho-peptide enrichment. High pH reversed phase fractionation and TiO2 based phospho-peptide enrichment was performed as reported previously [63]. Enriched phosphopeptides were run on a Proxeon nano nLC 1200 coupled to Q-Exactive HF. The samples were run on 105 minutes gradients, with MS1 and MS2 resolution of 60,000, isolation width of 0.8 m/z and normalized collision energy of 33. The raw data was analyzed using MaxQuant version 1.5.2.8 [64].

### Data and statistical analysis

Data were analyzed using GraphPad Prism version 6 (GraphPad Software). Results were presented as the standard deviation of the mean (SD, presented as error bars). Comparisons between experimental groups were made using unpaired 2-tailed Student *t* test. *P* < 0.05 was considered to be statistically significant.

## SUPPLEMENTARY MATERIALS FIGURES AND TABLES




