# Supplementary material for: STK3 is a therapeutic target for a subset of acute myeloid leukemias
Source: Oncotarget. 2018 May 22;9(39):25458–73. doi: 10.18632/oncotarget.25238 (PMC5986655; doi:10.18632/oncotarget.25238)
Supplement: Supplementary file 1 [file oncotarget-09-25458-s001.pdf]

STK3 is a therapeutic target for a subset of acute myeloid leukemias

SUPPLEMENTARY MATERIALS

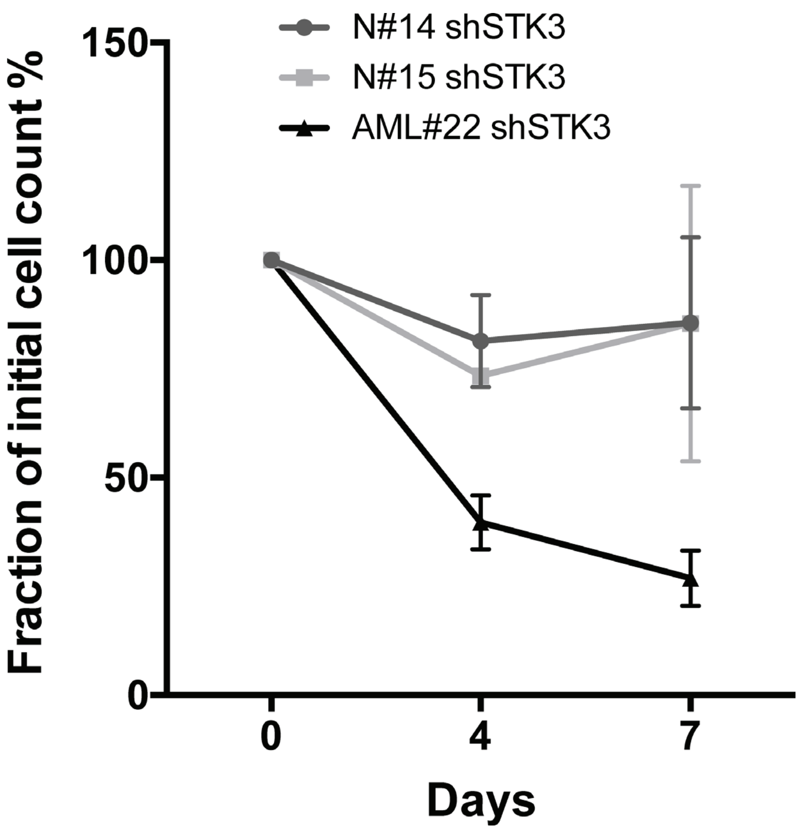

Supplemental Figure 1: Comparison of the impact of STK3 knock-down in AML#22 and two healthy donors. CD34+ hematopoietic progenitor cells were isolated from two healthy donors (N#14, N#15) and infected with lentiviral particles expressing the shRNA targeting STK3. Cell counts were performed at indicated time points. AML#22 cells served as positive control. All results were normalized to non-target control shRNA transduced cells to control for differential proliferation kinetics between the samples. Data are presented as mean ± SD.

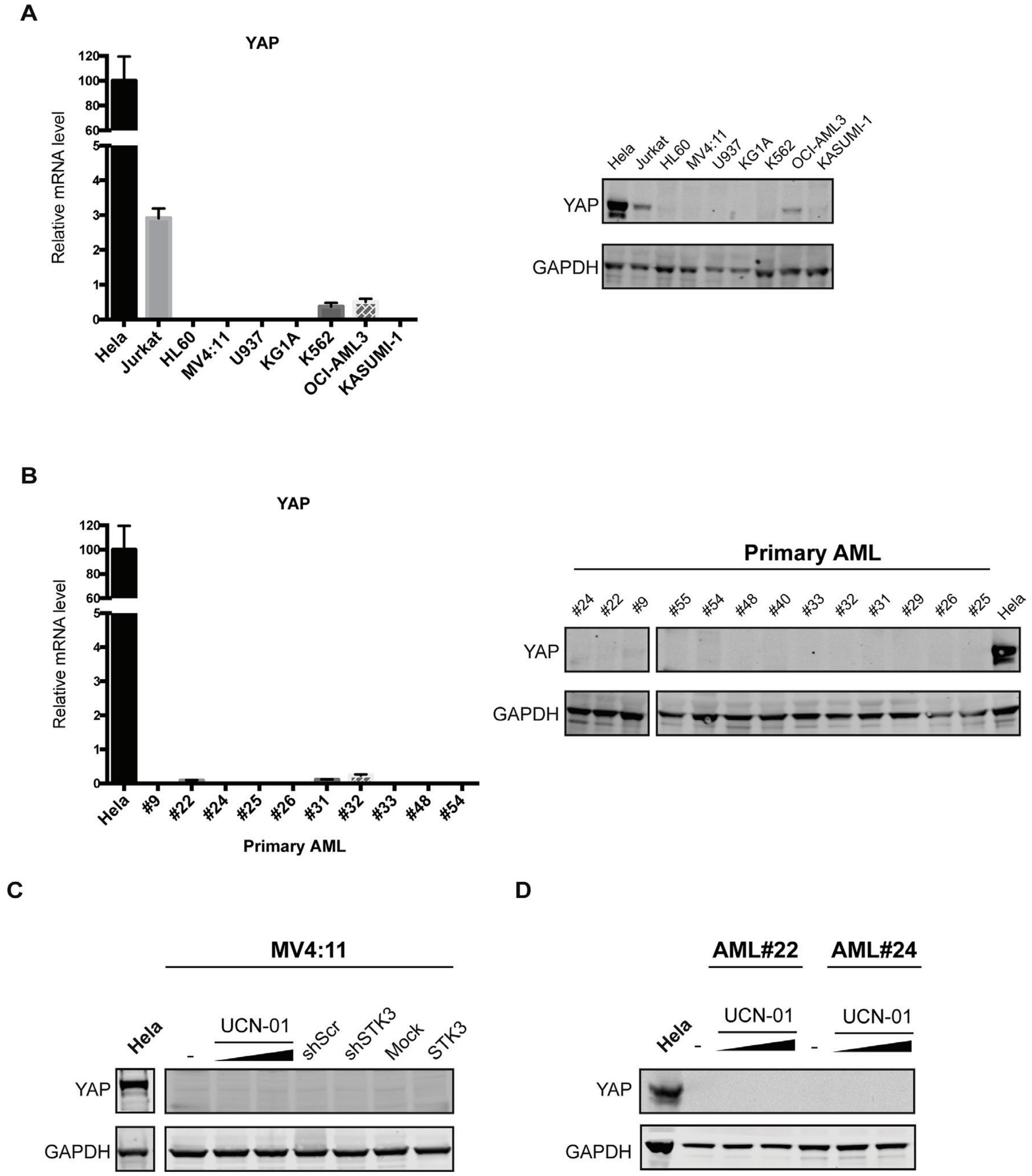

Supplemental Figure 2: Expression of YAP in AML cell lines and primary AML samples. (**A**) Basal YAP expression analyzed at mRNA levels by qPCR and at protein levels by western blot assay in indicated cell lines is shown. Data are presented as mean ± SD. (**B**) Basal YAP expression at mRNA levels and at protein levels analyzed in indicated primary AML cells is shown. Data are presented as mean ± SD. (**C**) YAP expression assessed by western blot after UCN-01 treatment, STK3 knock-down or STK3 over-expression in MV4:11 cells. (**D**) YAP expression analyzed in indicated primary AML cells after UCN-01 treatment. Hela cells were used as positive controls and GAPDH served as loading controls.

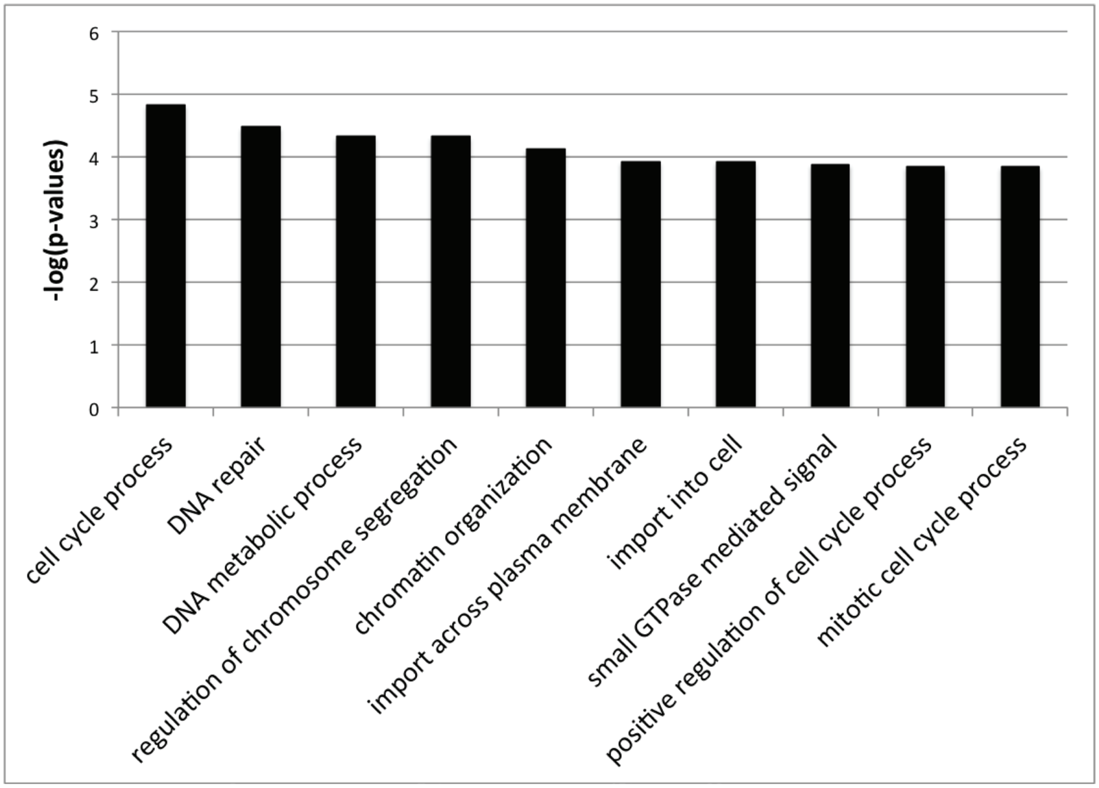

Supplemental Figure 3: Gene ontology (GO) analysis of differentially phosphorylated proteins after STK3 knock-down in MV4:11 cells. The most enriched cellular processes are highlighted. Note that the cell cycle pathway was the most enriched process.

Supplementary Table 1: A list of phosphopeptides quantified in cells infected with control shRNA and shRNA against STK3. See Supplementary_Table_1

Supplementary Table 2A: Human leukemia cell lines used in the study

Name	Age	Gender	Disease status	FAB	Model for mutations	Doubling time (h)	Reference
MV4:11	10	Male	At diagnosis	M5	MLL-AF4, FLT3	~50	Lange *et al.*,Blood (1987)
HL60	36	Female	At diagnosis	M2	NRAS, cMYC	~40	Collins SJ., Blood (1987)
MOLM13	20	Male	At relapse	M5	MLL-AF9, FLT3	~50	Matsuo *et al.*, Leukemia (1997)
OCI-AML3	57	Male	At diagnosis	M4	NPM1	~48	Quentmeier *et al.*, Leukemia (2005)
Kasumi-1	7	Male	At relapse	M2	AML1-ETO	~70	Asou *et al.*, Blood (1991)

Supplementary Table 2B: Human primary AML samples used in the study

sample ID	gender	patient age at sample collection	status at sample collection	cytogenetics	molecular genetics	disease at initial diagnosis
	[m/f]	[age]	[initial diagnosis/ relapse]			
AML2	f	67	initial diagnosis	Normal karyotype	*NPM*^{wt}, *FLT3*^{wt}	*de novo*
AML22	m	75	relapse	FISH i.O.	*NPM*^{mut}, *FLT3*^{wt}	*de novo*
AML24	m	71	initial diagnosis	n.a.	*NPM*^{wt}, *FLT3*^{wt}	*de novo*
AML28	m	64	initial diagnosis	Normal karyotype	*NPM*^{wt}, *FLT3*-ITD	*de novo*
AML29	f	58	initial diagnosis	Normal karyotype	*NPM*^{mut}, *FLT3*-ITD	*de novo*
AML31	f	54	initial diagnosis	del(5q)	*NPM*^{wt}, *FLT3*^{wt}	secondary AML
AML54	m	62	initial diagnosis	del(7q)	*NPM*^{wt}, *FLT3*^{wt}	*de novo*

Supplementary Table 3: shRNA and sgRNA sequences used in the study

RNAi or CRISPR-Cas9	Target	Sequence
shScr	human scramble control	CAACAAGATGAAGAGCACCAA
shSTK3	human STK3	GAATGCCAAACCTGTATCAAT
sgSTK3#1	human STK3	CGTTACCTCTTAGGCGCCGG
sgSTK3#2	human STK3	TGGTGAGGTTGCATTTCCTT
